# Enhanced prediction of breast cancer prognosis by evaluating expression of p53 and prostate-specific antigen in combination

**DOI:** 10.1038/sj.bjc.6690720

**Published:** 1999-10

**Authors:** H Yu, M A Levesque, G M Clark, E P Diamandis

**Affiliations:** Section of Cancer Prevention and Control, Feist-Weiller Cancer Center, Louisiana State University Medical Center, Shreveport, Louisiana, 71130, USA; Department of Pathology and Laboratory Medicine, Mount Sinai Hospital, Toronto, Ontario, M5G 1X5, Canada; Department of Laboratory Medicine and Pathobiology, University of Toronto, Toronto, Ontario, M5G 1L5, Canada; Department of Medicine, Division of Medical Oncology, San Antonio, Texas, 78284-7884, USA

**Keywords:** prostate-specific antigen, protein, p53, ELISA, breast neoplasms

## Abstract

p53 gene mutation is the most common genetic alteration in neoplastic diseases, including breast cancer, for which p53 alteration may indicate poor prognosis. Recent clinical evidence suggests that prostate-specific antigen (PSA) expression may identify breast cancer patients with favourable outcome. Assessment of p53 and PSA in combination, potentially offering improved prediction, has not yet been performed. Extracts from 952 primary breast carcinomas were assayed for PSA and p53 by quantitative enzyme-linked immunosorbent assays (ELISAs) developed by the authors. Concentrations of each marker were classified as negative or positive on the basis of median and 30th percentile cut-off points for p53 and PSA respectively. Patients followed for a median of 6 years having different combinations of negative or positive status for PSA and p53 were compared with respect to the relative risks (RRs) for relapse and death by Cox proportional hazards regression analysis, in which an interaction term was also evaluated, and with respect to disease-free survival (DFS) and overall survival (OS) probabilities by Kaplan–Meier plots and log-rank tests. Multivariate models were adjusted for oestrogen and progesterone receptor status, nodal status, patient age, tumour size, DNA ploidy, S phase fraction and receipt of chemotherapy. Interactions were not found between the status of PSA and p53 in the Cox models, in which PSA-negativity (RR = 1.47, *P* = 0.020 for DFS, and RR = 1.49, *P* = 0.023 for OS) and p53-positivity (RR = 1.46, *P* = 0.017 for DFS, and RR = 1.41, *P* = 0.033 for OS) were individually associated with prognosis. Evaluation of a combined three-level variable revealed that PSA(–)/p53(+) patients had significantly higher risks for relapse (RR = 2.13, *P* < 0.001) and death (RR = 2.08, *P* = 0.001) than PSA(+)/p53(–) patients, and that patients positive or negative for both markers had intermediate risks for the outcome events in the same multivariate analysis (RR = 1.45 for both DFS and OS). The results of our study demonstrate that the assessment of combined PSA and p53 expression status by ELISAs, easily applicable to breast tumour extracts prepared for steroid hormone receptor analyses, may stratify breast cancer patients into groups differing by relapse and death risks of greater magnitude than offered by the assessment of either p53 or PSA alone. © 1999 Cancer Research Campaign


*British Journal of Cancer* (1999) **81**(3), 490–495
© 1999 Cancer Research Campaign
Article no. bjoc.1999.0720

### Enhanced prediction of breast cancer prognosis by evaluating expression of p53 and prostate-specific antigen in combination

**H Yu[1], MA Levesque[2,3], GM Clark[4] and EP Diamandis[2,3]**

[1]Section of Cancer Prevention and Control, Feist-Weiller Cancer Center, Louisiana State University Medical Center, Shreveport, Louisiana 71130, USA; [2]Department of Pathology and Laboratory Medicine, Mount Sinai Hospital, Toronto, Ontario, Canada M5G 1X5; [3]Department of Laboratory Medicine and Pathobiology, University of Toronto, Toronto, Ontario, Canada M5G 1L5; [4]Department of Medicine, Division of Medical Oncology, University of Texas Health Science Center at San Antonio, San Antonio, Texas 78284-7884, USA

**Summary** p53 gene mutation is the most common genetic alteration in neoplastic diseases, including breast cancer, for which p53 alteration may indicate poor prognosis. Recent clinical evidence suggests that prostate-specific antigen (PSA) expression may identify breast cancer patients with favourable outcome. Assessment of p53 and PSA in combination, potentially offering improved prediction, has not yet been performed. Extracts from 952 primary breast carcinomas were assayed for PSA and p53 by quantitative enzyme-linked immunosorbent assays (ELISAs) developed by the authors. Concentrations of each marker were classified as negative or positive on the basis of median and 30th percentile cut-off points for p53 and PSA respectively. Patients followed for a median of 6 years having different combinations of negative or positive status for PSA and p53 were compared with respect to the relative risks (RRs) for relapse and death by Cox proportional hazards regression analysis, in which an interaction term was also evaluated, and with respect to disease-free survival (DFS) and overall survival (OS) probabilities by Kaplan–Meier plots and log-rank tests. Multivariate models were adjusted for oestrogen and progesterone receptor status, nodal status, patient age, tumour size, DNA ploidy, S phase fraction and receipt of chemotherapy. Interactions were not found between the status of PSA and p53 in the Cox models, in which PSA-negativity (RR = 1.47, $P = 0.020$ for DFS, and RR = 1.49, $P = 0.023$ for OS) and p53-positivity (RR = 1.46, $P = 0.017$ for DFS, and RR = 1.41, $P = 0.033$ for OS) were individually associated with prognosis. Evaluation of a combined three-level variable revealed that PSA(–)/p53(+) patients had significantly higher risks for relapse (RR = 2.13, $P < 0.001$) and death (RR = 2.08, $P = 0.001$) than PSA(+)/p53(–) patients, and that patients positive or negative for both markers had intermediate risks for the outcome events in the same multivariate analysis (RR = 1.45 for both DFS and OS). The results of our study demonstrate that the assessment of combined PSA and p53 expression status by ELISAs, easily applicable to breast tumour extracts prepared for steroid hormone receptor analyses, may stratify breast cancer patients into groups differing by relapse and death risks of greater magnitude than offered by the assessment of either p53 or PSA alone. © 1999 Cancer Research Campaign

**Keywords:** prostate-specific antigen; protein, p53; ELISA; breast neoplasms

Early administration of adjuvant therapy for breast cancer is intended to eliminate or suppress post-surgical residual disease to the extent that the onset of local recurrence or distant metastasis is delayed or prevented. Achievement of these goals depend, at least in part, on treatment being initiated soon after surgery and before the development of visible lesions. Needless and harmful treatment, however, must be avoided. Traditionally, decisions as to whether or not and how to treat breast cancer patients after surgery have been heavily contingent on the accuracy of estimating the behaviour and outcome of the disease, as well as of predicting the response of the disease to a specific therapeutic regimen (Gasparini et al, 1993).

Many clinical, histopathological and biochemical features of breast cancer have been demonstrated to have prognostic utility, although comparatively few of these markers have been shown to have value for predicting the success of adjuvant treatment. None of the numerous available prognostic markers, however, provides precise information on the disease outcome, especially for an individual patient. Furthermore, many of these markers do not provide prognostic information independent of other factors, and many are also difficult and costly to evaluate (McGuire and Clark, 1992; Gasparini et al, 1993). Improved capability of predicting breast cancer prognosis and response to treatment may likely be obtained through the identification of newer markers. Considering the complexity of cancer biology and the diversity of cancer cells, the application of new and existing markers in combination may also be an approach to facilitate accurate prediction.

Mutation of the p53 tumour suppressor gene occurs in approximately 20–50% of breast carcinomas (Soussi et al, 1994), and has been reported to be associated with unfavourable prognosis in this disease (Sjogren et al, 1996). In the majority of cases, the p53 mutation is predominantly mis-sense and leads to confirmational alteration of the protein, its accumulation in tumour cell nuclei, and consequently the utility of immunohistochemical staining methods or ELISAs for the detection of p53 protein as simple substitutes for mutational analyses (Thor et al, 1992; Allred et al, 1993; Borg et al, 1995, de Witte et al, 1996). Our recent study of a

*Received 7 December 1998*
*Revised 9 March 1999*
*Accepted 15 March 1999*

*Correspondence to:* EP Diamandis, Department of Pathology and Laboratory Medicine, Mount Sinai Hospital, 600 University Avenue, Toronto, Ontario, Canada M5G 1X5

large cohort of breast cancer patients also suggested that p53 protein levels in breast cancer extracts measured by an enzyme-linked immunosorbent assay (ELISA) were highly correlated with the risks of both relapse and death, and that these relationships were independent of other clinical and pathological features of the disease (Levesque et al, 1998).

PSA, a tissue specific protein used as a tumour marker for the diagnosis and management of prostate cancer (Partin and Oesterling, 1994), has been found to be present in a number of female tissues and body fluids (Pollen and Dreilinger, 1984; Pummer et al, 1992; Clements and Mukhtar, 1994; Diamandis et al, 1994, 1996; Yu and Diamandis, 1995*a*, 1995*b*; Yu et al, 1995*a*). Studies have shown that both normal and abnormal (including cancerous) breast tissues are capable of producing this protein (Yu et al, 1994*a*, 1996), expression of which is up-regulated by androgens and progestins through their receptors (Yu et al, 1994*b*; Zarghami et al, 1997), and that the presence of PSA in breast cancer may be indicative of a favourable prognosis (Yu et al, 1995*b*, 1998).

Because of the biological significance of p53 in cancer development and progression, as well as the substantial clinical applications of PSA in prostate cancer management, these molecules are two of the most extensively studied proteins in cancer research. Although previous studies have suggested potential clinical uses of each of these proteins for breast cancer prognosis, a study of their combined value for indicating patient prognosis has not yet been reported. In this study, the use of p53 and PSA in combination to predict the prognosis of breast cancer was prompted by observations that these biochemical markers apparently indicate different breast cancer prognoses, that they likely involve different aspects of tumour cell biology, and that their expression levels in breast tissue are not directly correlated. Using two highly sensitive and specific immunoassays, PSA and p53 concentrations in extracts of more than 900 breast cancer specimens were measured and assessed with respect to their prognostic values, individually and in combination using Cox regression survival analysis.

### MATERIALS AND METHODS

### Breast cancer specimens

The Ethics and Research Committee at the University of Toronto and the Institutional Review Board at the University of Texas Health Science Center at San Antonio each granted approval for this study. The collection of tumour tissue specimens and patient information has been described in detail elsewhere (Levesque et al, 1998; Yu et al, 1998) and is summarized briefly here. Tumour specimens from 1000 female breast cancer patients were obtained from the Breast Cancer Tissue Resource at the University of Texas Health Center at San Antonio, which maintains a large collection of leftover tissue specimens sent from 165 health care institutions throughout the USA to the Nichols Institute Research Laboratories for routine quantification of oestrogen receptors (ER) and progesterone receptors (PR) and for flow cytometric analyses of tumour cells. All patients included in the study had histologically confirmed diagnoses of primary breast carcinoma, had been operated between August 1985 and October 1991, and had been characterized for a number of clinical and pathological variables, including age at surgery, number of lymph nodes positive for malignancy, tumour size, ER and PR concentrations, DNA ploidy, S phase fraction, receipt of endocrine therapy, radiotherapy and chemotherapy, and survival times and outcomes. Of the patients studied, 97% underwent modified radical mastectomy with axillary lymph node dissection and 3% had incisional biopsy or lumpectomy with axillary lymph node dissection. Forty-five per cent of patients who had axillary lymph node dissection had positive nodes. The sizes of the tumours resected ranged from 0.1 to 14.5 cm and the median size was 2.3 cm. ER-positive or PR-positive lesions were found in 80% and 71% of the patients respectively. Flow cytometric analyses showed 31% of patients to have an S phase fraction above the cut-off level (≥ 6.7%) and 54% of patients to have an aneuploid DNA content. Patients who received post-surgical treatment included those who were administered regional radiotherapy alone (9%), adjuvant chemotherapy alone (16%), endocrine therapy alone (17%), both endocrine and radiation therapies (4%), both chemotherapy and radiotherapy (7%), both systemic adjuvant therapies (6%) and all three therapy types (2%). Patient follow-up times ranged from 7 to 121 months and had a median value of 73 months. The disease-free survival (DFS) time was defined as the time interval between the date of surgery and the date of the first documented evidence of local or regional axillary recurrence, distant metastasis, or new breast cancer. The overall survival (OS) time was defined as the interval between the date of surgery and the date of death due to any causes, or the date of last follow-up for those patients still alive at the termination of the study.

### Quantification of p53, PSA and other markers

The methods used for the preparation of tumour tissue extracts as well as for the measurements of p53 and PSA have been described elsewhere (Levesque et al, 1998; Yu et al, 1998). In brief, concentrations of p53 and PSA were measured with two time-resolved fluorometric immunoassays developed in our laboratory which employ two pairs of well-characterized antibodies in a sandwich configuration. The analytical performances of the assays, including precision, specificity and sensitivity, have been evaluated and documented previously (Levesque et al, 1995*b*; Ferguson et al, 1996). All specimens were measured in duplicate and the concentrations of each analyte were adjusted for the protein contents of the extracts, determined by a commercial kit (Pierce Chemical Co., Rockford, IL, USA). As described in detail elsewhere, ER and PR concentrations were determined using dual ligand binding assays (Dressler et al, 1988); cut-off levels for receptor positivity were 3 fmol $mg^{-1}$ and 5 fmol $mg^{-1}$ for ER and PR respectively (Clark and McGuire, 1983). The preparation of specimens and performance of flow cytometric assessments of the percentage of cells in S phase and the DNA content have also been reported elsewhere (Dressler et al, 1988). The cut-off value for S phase fraction considered a favourable prognostic indicator was 6.7% (Clark et al, 1989).

### Statistical analysis

The relationships between the expression status of p53 and PSA and patient survival were assessed using Cox proportional hazards regression models of DFS and OS (Cox, 1972). PSA and p53 were evaluated dichotomously in the models using cut-off points equal to the 30th and 50th percentiles of the distributions of PSA and p53 respectively. Selections of these cut-offs were based on the individual prognostic values of each of these markers in our previous studies (Levesque et al, 1998; Yu et al, 1998). PSA(+)

© 1999 Cancer Research Campaign 

**Table 1** Univariate associations of expression status of prostate specific antigen (PSA) and p53 with disease-free and overall survival[a]

| Variable status[b] | Number | RR | 95% CI | P-value |
|---|---|---|---|---|
| Unadjusted disease-free survival (*n* = 952) | | | | |
| PSA(+) | 626 | 1.00 | | |
| PSA(–) | 326 | 1.20 | 1.04–1.41 | 0.014 |
| p53(–) | 476 | 1.00 | | |
| p53(+) | 476 | 1.19 | 1.03–1.37 | 0.017 |
| PSA(+)p53(–) | 364 | 1.00 | | |
| PSA(±)p53(±)[c] | 460 | 1.43 | 1.18–1.75 | |
| PSA(–)p53(+) | 128 | 2.04 | 1.39–3.13 | <0.001 |
| Unadjusted overall survival (*n* = 952) | | | | |
| PSA(+) | 626 | 1.00 | | |
| PSA(–) | 326 | 1.18 | 0.99–1.39 | 0.059 |
| p53(–) | 476 | 1.00 | | |
| p53(+) | 476 | 1.18 | 1.02–1.36 | 0.028 |
| PSA(+)p53(–) | 364 | 1.00 | | |
| PSA(±)p53(±)[c] | 460 | 1.39 | 1.11–1.72 | |
| PSA(–)p53(+) | 128 | 1.92 | 1.25–2.94 | 0.003 |

[a]RR = relative risk estimated using the Cox proportional hazard regression model; CI = confidence interval; *P*-values are two-sided. [b]Cut-off points for PSA- and p53-positivity were 1 ng $g^{-1}$ and 0.16 μg $g^{-1}$ respectively. [c]Indicates either PSA(+)p53(+) or PSA(–)p53(–).

**Table 2** Multivariate associations of expression status of prostate specific antigen (PSA) and p53 with disease-free and overall survival[a]

| Variable status[b] | Number | RR | 95% CI | P-value |
|---|---|---|---|---|
| Adjusted disease-free survival (*n* = 838)[c] | | | | |
| PSA(+) | 633 | 1.00 | | |
| PSA(–) | 205 | 1.47 | 1.06–2.00 | 0.020 |
| p53(–) | 408 | 1.00 | | |
| p53(+) | 430 | 1.46 | 1.08–1.97 | 0.017 |
| PSA(+)p53(–) | 319 | 1.00 | | |
| PSA(±)p53(±)[d] | 403 | 1.45 | 1.18–1.82 | |
| PSA(–)p53(+) | 116 | 2.13 | 1.39–3.23 | <0.001 |
| Adjusted overall survival (*n* = 838)[c] | | | | |
| PSA(+) | 633 | 1.00 | | |
| PSA(–) | 205 | 1.49 | 1.05–2.13 | 0.023 |
| p53(–) | 408 | 1.00 | | |
| p53(+) | 430 | 1.41 | 1.03–1.93 | 0.033 |
| PSA(+)p53(–) | 319 | 1.00 | | |
| PSA(±)p53(±)[d] | 403 | 1.45 | 1.15–1.82 | |
| PSA(–)p53(+) | 116 | 2.08 | 1.33–3.33 | 0.001 |

[a]RR = relative risk estimated using the Cox proportional hazard regression model; CI = confidence interval; *P*-values are two-sided. [b]Cut-off points for PSA- and p53-positivity were 1 ng $g^{-1}$ and 0.16 μg $g^{-1}$ respectively. [c]Multivariate analysis adjusted for age, nodal status, tumour size, DNA ploidy, S phase fraction, oestrogen receptor status, progesterone receptor status, and receipt of endocrine therapy, chemotherapy and radiotherapy. [d]Indicates either PSA(+)p53(+) or PSA(–)p53(–).

patients and p53(–) patients served as the reference groups for the calculation of the relative risks (RRs). Interactions between PSA and p53 were also examined in the Cox regression models, which contained the dichotomous PSA and p53 variables in addition to their product term. This term was created by multiplying the PSA and p53 variables, when each variable was coded as either –1 or +1 on the basis of the cut-off points defined previously, resulting in a two-category interaction term also having values of –1 or +1. Correlation between concentrations of p53 and PSA were evaluated by the calculation of the Spearman correlation coefficient.

In order to evaluate the combined prognostic value of PSA and p53 in comparison to their individual values in prognosis, a three-level ordinal variable was created using the combined categories of PSA and p53. The first level of this new variable consisted of patients whose tumour extracts were both PSA(+) and p53(–). The second level included patients whose tumours were either positive for both markers, i.e. PSA(+) and p53(+), or negative for both, i.e. PSA(–) and p53(–). Patients in the third level had tumours which were both PSA(–) and p53(+). The Cox regression models were developed at both univariate and multivariate levels in order to evaluate the independent impact of the combined PSA and p53 variable, on DFS and OS. Only patients for whom complete information was collected were included in the multivariate analysis, which incorporated the following covariables: age at surgery, nodal status, tumour size, ER status, PR status, DNA ploidy, S phase fraction and post-operative treatment, including endocrine therapy, chemotherapy and radiation therapy. Kaplan–Meier survival curves were also constructed to demonstrate the effects of PSA and p53 in combination on DFS and OS probabilities (Kaplan and Meier, 1958), differences over time for which were evaluated using the log-rank test (Mantel, 1966).

### RESULTS

The concentrations of PSA and p53 in the 952 breast tumour extracts for which sufficient volume permitted assay of both proteins varied widely, and the frequency distributions of the data were extremely skewed towards the lower values for both of the variables. The mean, standard deviation (s.d.) and median of the PSA concentrations ranging from 0 to 19 629 ng $g^{-1}$ were 150.1 ng $g^{-1}$, 1078 ng $g^{-1}$, and 6.0 ng $g^{-1}$ respectively. The concentrations of p53 ranged from 0 to 58.02 μg $g^{-1}$ and were distributed with a mean of 1.28 μg $g^{-1}$, a s.d. of 4.52 μg $g^{-1}$ and a median of 0.17 μg $g^{-1}$. Correlation between p53 and PSA concentrations was not evident ($r$ = –0.016, $P$ = 0.271).

As demonstrated in our previous study (Yu et al, 1998), PSA used as a categorical variable was associated inversely with tumour size ($P$ = 0.032), S phase fraction ($P$ = 0.002) and DNA aneuploidy ($P$ = 0.004) in contingency tables analysed by $\chi^2$ tests; no association was found between PSA and patient age ($P$ = 0.517), nodal status ($P$ = 0.393) and steroid receptor status ($P$ = 0.72 for ER and $P$ = 0.28 for PR) in the same analyses. With respect to p53 protein status (Levesque et al, 1998) considered in relation to the same variables, a positive association was found with S phase fraction ($P$ = 0.04) and DNA aneuploidy ($P$ = 0.005), whereas ER and PR were inversely associated with p53-positive tumours ($P$ = 0.003 for ER and $P$ = 0.05 for PR). Patient age ($P$ = 0.13), tumour size ($P$ = 0.19) and nodal status ($P$ = 0.63) were not associated with p53 protein status.

The Cox regression models were developed initially with two separate variables, PSA and p53, and both were statistically significant in the models; for PSA-negativity, the RRs were 1.20 ($P$ = 0.01) for relapse and 1.18 ($P$ = 0.06) for death, whereas for p53-positivity, the RRs were 1.19 ($P$ = 0.02) for relapse and 1.18 ($P$ = 0.03) for death. These results, shown in Table 1, demonstrate 20% and 18% increases in risks for relapse and death, respectively, associated with tumour extract PSA levels below the cut-off point for positivity. p53-positivity was similarly associated with a 19% increased risk for relapse and an 18% increased risk for death, relative to p53-negative tumours. Introduction of the interaction

 © 1999 Cancer Research Campaign

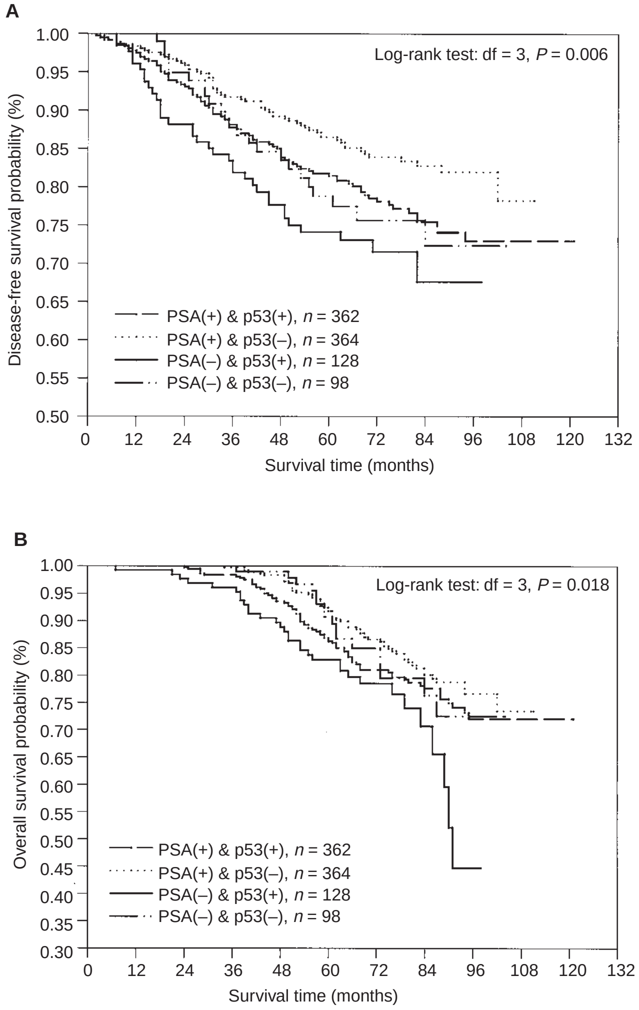

**Figure 1** Kaplan–Meier disease-free (**A**) and overall (**B**) survival analysis of breast cancer patients divided into four groups based on the expression status of both p53 and prostate specific antigen (PSA) proteins. p53 status and PSA status were based upon cut-off points equal to the median (0.16 µg $g^{-1}$), and the 30th percentile (1 ng $g^{-1}$), of the frequency distributions of p53 and PSA concentrations respectively term, which was not statistically significant when evaluated alone in the models ($P$ = 0.66 for DFS and $P$ = 0.46 for OS), into the Cox analysis resulted in very little reduction in the random variation attributed to the models. Substantial changes in the risks of relapse and death were indicated when the two markers were combined together in the analysis (Table 1). The maximum risk differences were approximately 100% when the comparison was made between the two extreme groups, i.e. comparing patients with the anticipated best prognosis [PSA(+) and p53(–)] with those having the worst expected outcome [PSA(–) and p53(+)]. The RR for relapse was 2.04 ($P$ < 0.001), and the RR for death was 1.92 ($P$ = 0.003). Patients in the other group who were either negative or positive for both markers had intermediate risks for relapse (RR = 1.43) and death (RR = 1.39).

Figures 1 and 2 show the Kaplan–Meier DFS and OS curves among the four groups of patients having different combinations of positivity status for PSA and p53, demonstrating similar differences in survival as observed in the Cox regression analysis. Evident from the Figures are that patients whose tumour extracts were positive for PSA and simultaneously negative for p53 had survival probabilities which were consistently the highest over time, and that patients whose tumours were PSA-negative and p53-positive had consistently the lowest survival rates. The difference in survival probability between the two extreme groups was more evident in terms of DFS than OS. The survival probabilities for patients in the two groups with negative or positive status for both markers did not differ significantly.

Shown in Table 2 are the results of the Cox regression analysis of DFS and OS in which risks for each outcome were adjusted for several clinical and pathological variables. The RRs for relapse and death of the combined PSA and p53 variable were not altered markedly after their adjustment in the multivariate analysis, in that the twofold statistically significant differences in risks of relapse and death between the two extreme groups were maintained. However, the RRs for the individual PSA and p53 variables were increased in magnitude after the adjustment. The risks of relapse and death for PSA-negative patients were almost 50% higher compared to PSA-positive patients. The p53-positive patients had more than 40% increased risks of relapse and death in comparison to p53-negative patients. Both estimates of RR were statistically significant.

In the multivariate analysis, variables which were also statistically significant in the Cox regression model were nodal status (RR = 1.52, 95% confidence intervals (CI) = 1.25–1.84, $P$ < 0.001 for DFS, and RR = 1.69, 95% CI 1.40–2.04, $P$ < 0.001 for OS) and tumour size (RR = 1.38, 95% CI 1.00–1.89, $P$ = 0.047 for DFS, and RR = 1.43, 95% CI 1.02–1.98, $P$ = 0.036 for OS). Patients who received chemotherapy also showed a significantly increased risk of relapse in the multivariate model (RR = 1.76, 95% CI 1.28–2.42, $P$ < 0.001), a finding which was not seen in OS analysis and was likely due to the selection of patients exhibiting local recurrence or metastasis for receipt of chemotherapy. None of the steroid hormone receptors, or DNA ploidy or the S phase fraction were shown to be independent prognostic factors in the multivariate analysis (data not shown).

### DISCUSSION

No study has yet evaluated PSA and p53 in combination for breast cancer prognosis. The concept of using these proteins together to predict the survival outcome of breast cancer was based on findings that PSA and p53 expression indicated favourable and unfavourable prognoses, respectively, and that improved prediction may be offered by considering them together. Further support for this notion is provided by the absence of known functional associations between the proteins, and the lack of statistical interaction between them in relation to patient survival.

Much greater differences in risks of relapse and death were observed when PSA and p53 were utilized in combination compared to use of the two markers individually. Furthermore, adjustment of the associations with other variables showed little impact on the risks for relapse and death conferred by using the combined variable of PSA and p53. However, PSA and p53 used separately in the survival analysis were shown substantial changes when the relationships were adjusted for other clinical and pathological factors. This observation suggests that use of these markers in combination in survival analysis generates more consistent and reliable results for prognosis than use of the markers individually.

*© 1999 Cancer Research Campaign*

There is presently no experimental evidence implying a direct biological link between PSA and p53. Comparison of the levels of the two proteins in breast cancer tissues in our preliminary study did not find any indication that their tissue concentrations were correlated to each other (Levesque et al, 1995*a*) – a finding confirmed in the present study. However, a possible indirect relationship between PSA and p53 has been suggested with respect to one of the insulin-like growth factor binding proteins, IGFBP-3. It has been shown that wild-type p53 can transcriptionally up-regulate expression of IGFBP-3 (Buckbinder et al, 1995), which can serve as a substrate for proteolysis in seminal plasma by PSA (Cohen et al, 1992).

PSA is a glycoprotein which, until recently, has been believed to be produced and secreted exclusively by the epithelial cells of the prostate, and therefore has been used widely as a tumour marker in the diagnosis and management of prostate cancer (Partin and Oesterling, 1994). The expression of PSA has been found to be present in a number of female organs (Pollen and Dreilinger, 1984; Pummer et al, 1992; Clements and Mukhtar, 1994; Diamandis et al, 1994, 1996; Yu and Diamandis, 1995*a*, 1995*b*; Yu et al, 1995*a*) including breast tissue (Yu et al, 1994*a*, 1996), in which its production has been shown to be up-regulated by androgens and progestins via their specific receptors (Yu et al, 1994*b*; Zarghami et al, 1997). Moreover, in a cell culture system, oestrogens were demonstrated to impair the PSA production induced by androgens (Yu et al, 1994*b*). In a small-scale clinical study, PSA status in extracts of breast tumours was shown to be associated with the risk of breast cancer relapse (Yu et al, 1995*b*). This finding has been confirmed and extended in a large cohort study (Yu et al, 1998), in which we observed that the risks of both relapse and death were significantly reduced in patients with PSA-positive breast cancer compared to those with PSA-negative lesions, and these associations were independent of other prognostic indicators, such as nodal status, tumour size and steroid hormone receptor status.

Both the physiological function of PSA in breast tissue, and the cellular basis for the favourable prognosis associated with its expression, remain unknown. Based on in vitro observations of breast cancer cell lines that the PSA gene is regulated by several steroid hormones through their receptors (Yu et al, 1994*b*; Zarghami et al, 1997), we have speculated that the presence of PSA could indicate either the preservation of a steroid regulatory pathway in well-differentiated cancer cells, or the suppression of an oestrogenic impact on these cells by androgen (Yu et al, 1995*b*). However, our observations also invoke a possible role for PSA in growth factor regulation (Oesterling et al, 1989; Cohen et al, 1992, 1994).

The important biological functions of p53 in cell cycle regulation and programmed cell death have been well established (Hartwell and Kastan, 1994; Lane, 1994; Levine et al, 1994). Loss of these cellular functions consequent to p53 gene mutation has been considered to be highly relevant to cancer development and progression (Chang et al, 1993; Greenblatt et al, 1994; Karp and Broder, 1995) and occurs in 20–50% of breast cancers (Soussi et al, 1994). Breast cancer patients with p53 mutation have been shown to be more likely to develop relapse or to die compared to those without p53 mutation (Sjogren et al, 1996). Although findings from many studies have suggested potential clinical implications of the detection of p53 gene mutations, the implementation of mutational analysis for p53 remains rather complex and costly. Because mis-sense p53 gene mutation, accounting for over 80% of all p53 genetic abnormalities, typically results in p53 protein accumulation in tumour cell nuclei, the detection of p53 protein by immunochemical methods has been shown to be highly correlated with mutation of the p53 gene. This method has therefore been considered a simple and rapid alternative to the assessment of p53 gene mutational status by molecular biology techniques (Thor et al, 1992; Allred at al, 1993; de Witte et al, 1996). The validity of immunohistochemical detection of p53 protein is further supported by findings of studies using ELISA to measure the protein (Borg et al, 1995; de Witte et al, 1996). A recent study by our group also demonstrated that the risks of relapse or death were elevated significantly with increased concentrations of p53 protein in extracts of breast carcinomas and that this dose–response relationship was independent of other prognostic indicators (Levesque et al, 1998). Although requiring less skilled labour and interpretation than the detection of p53 gene alterations by mutational analysis, immunostaining for p53 protein remains arguably more subjective and qualitative than ELISA, which may yield more reproducible and inherently quantitative results (Diamandis and Levesque, 1995). Furthermore, a direct comparison of ELISA-determined p53 concentrations in tumour extracts with the mutational status of the p53 gene, determined by DNA sequencing of p53 exons 5–9 in a series of ovarian carcinomas, has been recently performed by our group and has revealed the general concordance between mis-sense point mutation in the core domain and overexpression of p53 protein (Lianidou et al, 1999).

From a clinical perspective, ELISA-type immunoassays for the evaluation of p53 and PSA may offer a number of strengths over immunohistochemistry. In practice, ELISAs may more easily process a large number of specimens with short assay time and at a low cost. Furthermore, results from ELISAs are more reproducible and objective. Although ELISAs require the preparation of soluble extracts of tissue specimens of greater mass than needed for sectioning and immunostaining, and provides no information on the expression of the protein in the context of intracellular or histological features, these limitations are offset by the quantitative nature of the results and the potentially smaller bias in tissue sampling. Moreover, the adoption of ELISA methods for p53 detection by clinical laboratories would be facilitated further by the present availability of cytosolic extracts of breast tumours prepared for routine ER and PR determinations.

In summary, by evaluating the prognostic value of ELISA-quantified PSA and p53 in combination in more than 900 patients with breast cancer, we conclude that the determination of concentrations of both proteins in breast tumour extracts provides superior prognostic information than assessment of the individual markers. Studies in other patient populations, however, especially those with well-defined post-operative treatment, are needed to confirm our finding.

### ACKNOWLEDGEMENTS

This work was supported by a grant to Dr Eleftherios P Diamandis from the Canadian Breast Cancer Research Initiative (CBCRI) of the National Cancer Institute of Canada.

### REFERENCES

Allred DC, Clark GM, Elledge R, Fuqua SAW, Brown RW, Chamness GC, Osborne CK and McGuire WL (1993) Association of p53 protein expression with tumor cell proliferation rate and clinical outcome in node-negative breast cancer. *J Natl Cancer Inst* **85**: 200–206

Borg A, Lennerstrand J, Stenmark-Askmalm M, Ferno M, Brisfors A, Ohrvik A, Stal O, Killander D, Lane D and Brundell J (1995) Prognostic significance of p53

© 1999 Cancer Research Campaign overexpression in primary breast cancer; a novel luminometric immunoassay applicable on steroid receptor cytosols. *Br J Cancer* **71**: 1013–1017

Buckbinder L, Talbott R, Velasco-Miguel S, Takenaka I, Faha B, Seizinger BR and Kley N (1995) Induction of the growth inhibitor IGF-binding protein 3 by p53. *Nature* **377**: 646–649

Chang F, Syrjanen, Tervahauta A and Syrjanen K (1993) Tumorigenesis associated with the p53 tumour suppressor gene. *Br J Cancer* **68**: 653–661

Clark GM and McGuire WL (1983) Progesterone receptors and human breast cancer. *Breast Cancer Res Treat* **3**: 157–163

Clark GM, Dressler LG, Owens MA, Pounds G, Oldaker T and McGuire WL (1989) Prediction of relapse or survival in patients with node-negative breast cancer by DNA flow cytometry. *N Engl J Med* **320**: 627–633

Clements J and Mukhtar A (1994) Glandular kallikreins and prostate specific antigen are expressed in the human endometrium. *J Clin Endocrinol Metab* **78**: 1536–1539

Cohen P, Graves HCB, Peehl DM, Kamarel M, Giudice LC and Rosenfeld RG (1992) Prostate-specific antigen (PSA) is an insulin-like growth factor binding protein-3 protease found in seminal plasma. *J Clin Endocrinol Metab* **75**: 1046–1053

Cohen P, Peehl DM, Graves HCB and Rosenfeld RG (1994) Biological effects of prostate specific antigen as an insulin-like growth factor-binding-3 protease. *J Endocrinol* **142**: 407–415

Cox DR (1972) Regression models and life tables. *J R Stat Soc B* **34**: 187–202

de Witte HH, Koekens JA, Lennerstrand J, Smid M, Look MP, Klijn JGM, Benraad TJ and Berns EMJJ (1996) Prognostic significance of TP53 accumulation in human primary breast cancer: comparison between a rapid quantitative immunoassay and SSCP analysis. *Int J Cancer* **69**: 125–130

Diamandis EP and Levesque M (1995) Assessment of p53 protein overexpression by non-immunohistochemical methods. *J Pathol* **175**: 93–94

Diamandis EP, Yu H and Sutherland DJA (1994) Detection of prostate specific antigen immunoreactivity in breast tumors. *Breast Cancer Res Treat* **32**: 291–300

Diamandis EP, Yu H and Lopez-Otin C (1996) Prostate specific antigen – a new constituent of breast cyst fluid. *Breast Cancer Res Treat* **38**: 259–264

Dressler LG, Seamer LC, Owens MA, Clark GM and McGuire WL (1988) DNA flow cytometry and prognostic factors in 1131 frozen breast cancer specimens. *Cancer* **61**: 420–427

Ferguson RA, Yu H, Kalyvas M, Zammit S and Diamandis EP (1996) Ultrasensitive detection of prostate specific antigen by a new time resolved immunofluorometric assay and the Immulite immunochemiluminescent third-generation assay: potential applications in prostate and breast cancers. *Clin Chem* **42**: 675–684

Gasparini G, Pozza F and Harris AL (1993) Evaluating the potential usefulness of new prognostic and predictive indicators in node-negative breast cancer patients. *J Natl Cancer Inst* **85**: 1206–1219

Greenblatt MS, Bennett WP, Hollstein M and Harris CC (1994) Mutations in the p53 tumor suppressor gene: clues to cancer etiology and molecular pathogenesis. *Cancer Res* **54**: 4855–4878

Hartwell LH and Kastan MB (1994) Cell cycle control and cancer. *Science* **266**: 1821–1828

Kaplan EL and Meier P (1958) Nonparametric estimation from incomplete observations. *J Am Stat Assoc* **53**: 457–481

Karp JE and Broder S (1995) Molecular foundations of cancer: new targets for intervention. *Nature Med* **1**: 309–320

Lane DP (1994) The regulation of p53 function. *Int J Cancer* **57**: 623–627

Levesque MA, Clark GM, Yu H and Diamandis EP (1995*a*) Immunofluorometric analysis of p53 protein and prostate-specific antigen in breast tumours and their association with other prognostic indicators. *Br J Cancer* **72**: 720–727

Levesque MA, D'Costa M, Angelopoulou K and Diamandis EP (1995*b*) Time-resolved immunofluorometric assay of p53. *Clin Chem* **41**: 1720–1729

Levesque MA, Yu H, Clark GM and Diamandis EP (1998) ELISA-detected p53 protein accumulation: a prognostic factor in a large breast cancer cohort. *J Clin Oncol* **16**: 2641–2650

Levine AJ, Perry ME, Chang A, Silver A, Dittmer D, Wu M and Welsh D (1994) The role of the p53 tumour-suppressor gene in tumorigenesis. *Br J Cancer* **69**: 409–416

Lianidou ES, Levesque MA, Katsaros D, Angelopoulou K, Yu H, Genta F, Arisio R, Massobrio M, Bharaj B and Diamandis EP (1999) Immunofluorometric assay of p53 protein versus sequencing of p53 exons 5 to 9 for the detection of p53 abnormalities in ovarian carcinoma. *Anticancer Res* **19**: 749–756

Mantel N (1966) Evaluation of survival data and two new rank order statistics arising in its consideration. *Cancer Chemother Rep* **50**: 163–170

McGuire WL and Clark GM (1992) Prognostic factors and treatment decisions in axillary-node-negative breast cancer. *N Engl J Med* **326**: 1756–1761

Oesterling JE (1989) Prostate specific antigen: a critical assessment of the most useful tumor marker for adenocarcinoma of the prostate. *J Urol* **145**: 907–923

Partin AW and Oesterling JE (1994) The clinical usefulness of prostate specific antigen: update 1994. *J Urol* **152**: 1358–1368

Pollen JJ and Dreilinger A (1984) Immunohistochemical identification of prostatic acid phosphatase and prostate specific antigen in female periurethral gland. *Urology* **23**: 303–304

Pummer K, Wirnsberger G, Purstner P, Stettner H and Wandschneider G (1992) False positive prostate specific antigen values in the sera of women with renal cell carcinoma. *J Urol* **148**: 21–23

Sjögren S, Inganas M, Norberg T, Lindgren A, Nordgren H, Holmberg L and Bergh J (1996) The p53 gene in breast cancer: prognostic value of complementary DNA sequencing versus immunohistochemistry. *J Natl Cancer Inst* **88**: 173–182

Soussi T, Legros Y, Lubin R, Ory K and Schlichtholz (1994) Multifactorial analysis of p53 alteration in human cancer: a review. *Int J Cancer* **57**: 1–9

Thor AD, Moore DH, Edgerton SM, Kawasaki ES, Reihsaus E, Lynch HT, Marcus JN, Schwartz L, Chen LC, Mayall BH and Smith HS (1992) Accumulation of p53 tumor suppressor gene protein: an independent marker of prognosis in breast cancer. *J Natl Cancer Inst* **84**: 845–855

Yu H and Diamandis EP (1995*a*) Prostate specific antigen immunoreactivity in amniotic fluid. *Clin Chem* **41**: 204–210

Yu H and Diamandis EP (1995*b*) Prostate specific antigen in milk of lactating women. *Clin Chem* **41**: 54–60

Yu H, Diamandis EP and Sutherland DJA (1994*a*) Immunoreactive prostate specific antigen levels in female and male breast tumors and its association with steroid hormone receptors and patients age. *Clin Biochem* **27**: 75–79

Yu H, Diamandis EP, Zarghami N and Grass L (1994*b*) Induction of prostate specific antigen production by steroids and tamoxifen in the breast cancer cell lines. *Breast Cancer Res Treat* **32**: 301–310

Yu H, Diamandis EP, Levesque MA, Asa SL, Monne M and Croce CM (1995*a*) Expression of the prostate-specific antigen gene by a primary ovarian carcinoma. *Cancer Res* **55**: 1603–1606

Yu H, Giai M, Diamandis EP, Katsaros D, Sutherland DJA, Levesque MA, Roagna R, Ponzone R and Sismondi P (1995*b*) Prostate-specific antigen is a new favorable prognostic indicator for women with breast cancer. *Cancer Res* **55**: 2104–2110

Yu H, Diamandis EP, Levesque M, Giai M, Roagna R, Ponzone R, Sismondi P, Monne M and Croce M (1996) Prostate specific antigen in breast cancer, benign breast disease and normal breast tissue. *Breast Cancer Res Treat* **40**: 171–178

Yu H, Levesque MA, Clark GM and Diamandis EP (1998) Prognostic value of prostate specific antigen for women with breast cancer – a large cohort study. *Clin Cancer Res* **4**: 1489–1497

Zarghami N, Grass L and Diamandis EP (1997) Steroid hormone regulation of prostate specific antigen gene expression in breast cancer. *Br J Cancer* **75**: 579–588

*© 1999 Cancer Research Campaign*
